# *Cladanthus scariosus* Essential Oil and Its Principal Constituents with Cytotoxic Effects on Human Tumor Cell Lines

**DOI:** 10.3390/plants13111555

**Published:** 2024-06-04

**Authors:** Natale Badalamenti, Vincenzo Ilardi, Maurizio Bruno, Filippo Maggi, Luana Quassinti, Massimo Bramucci

**Affiliations:** 1 Department of Biological, Chemical and Pharmaceutical Sciences and Technologies (STEBICEF), Università degli Studi di Palermo, 90128 Palermo, Italy; vincenzo.ilardi@unipa.it (V.I.); maurizio.bruno@unipa.it (M.B.); 2 NBFC—National Biodiversity Future Center, 90133 Palermo, Italy; 3 Chemistry Interdisciplinary Project (ChIP) Research Center, School of Pharmacy, University of Camerino, 62032 Camerino, Italy; luana.quassinti@unicam.it (L.Q.); massimo.bramucci@unicam.it (M.B.)

**Keywords:** Asteraceae, breast adenocarcinoma cell, *Cladanthus scariosus*, colon adenocarcinoma cell, germacrene D, malignant melanoma cell

## Abstract

*Cladanthus* is a small genus of the Asteraceae family comprising just five species that, apart from *Cladanthus mixtus* (L.) Chevall., has a large distribution in all the Mediterranean countries, mainly in the North Africa area. Several ethnopharmacological uses have been reported for species of this genus. Notably, *Cladanthus scariosus* (Ball) Oberpr. & Vogt is endemic to Morocco. Seeking to delve deeper into the phytochemistry and pharmacological aspects of this species, in this work, we investigated the essential oil (EO) obtained from the aerial parts of a locally sourced accession, hitherto unexplored, growing wild near Tizi n’Ticha, Morocco. The chemical composition of the EO, obtained by the hydrodistillation method, was evaluated by GC and GC-MS. The most abundant EO constituent was germacrene D (13.2%), the principal representative of the sesquiterpene hydrocarbons class (27.2%). However, the major class of constituents was monoterpene hydrocarbons (43.0%), with *α*-pinene (11.9%), sabinene (10.2%), *p*-cymene (8.5%), and *α*-phellandrene (5.2%) as the most abundant. The EO and its main constituents have been tested for their possible cytotoxic activity against three human tumor cell lines (MDA-MB 231, A375, and CaCo2) using the MTT assay, with corresponding IC_50_ values of 13.69, 13.21, and 22.71 µg/mL, respectively. Germacrene D and terpinen-4-ol were found to be the most active constituents with IC_50_ values between 3.21 and 9.53 µg/mL. The results demonstrate remarkable cytotoxic activity against the three human tumor cell lines studied, and in the future, further analyses could demonstrate the excellent potential of *C. scariosus* EO as an antitumor agent.

## 1. Introduction

The genus *Cladanthus* Cass. is a genus with wide distribution in the Mediterranean basin [1]. *Cladanthus arabicus* (L.) Cass., which provides the type of *Cladanthus*, was enclosed in *Chamaemelum* sect. *Santolinopsis* Benedí. This section also included *Ch. eriolepis* (Maire) Benedí, *Ch. flahaultii* (Emb.) Benedí, *Ch. mixtum* (L.) All., and *Ch. scariosum* (Ball) Benedí, which showed morphological characters (floret and achene) very similar to *C. arabicus*. On the other hand, no phylogenetic relationship with the taxa of the *Chamaemelum* sect. *Chamaemelum* (consisting of *Ch. fuscatum* (Brot.) Vasc. and *Ch. nobile* (L.) All) were observed. Consequently, the genus *Cladanthus* (a name that has priority over *Ormenis* Cass., based on *Anthemis mixta* L.) was considered distinct from *Chamaemelum* [2].

Actually, five species are accepted in the genus *Cladanthus* [3] and their distribution as well as their synonymous variants are reported in Table 1.

Several ethnopharmacological uses have been reported for the species of this genus. *C. eriolepis* is endemic to Morocco and used by the local population against respiratory problems and as a poultice against bee stings [4,5]. *C. eriolepis* is also used to heal various health conditions including gastrointestinal disorders, stomach ulcers, hypertension, and helminthiasis [6]. *C. arabicus*, instead, is used in the treatment of diabetes in the High Atlas Central of Morocco [7] and of icterus [8]. *C. mixtus*, known as Moroccan chamomile, is very abundant in the Gharb region [9,10,11], and the collected plants are used for the extraction of an essential oil used in perfumery and cosmetics, with Morocco being the only supplier of this product on the international market [9]. Moroccan chamomile leaves and flowers have been largely used in traditional Moroccan medicine as an infusion to treat various ailments as an analgesic, antiallergic, anti-inflammatory, antispasmodic, carminative, digestive, febrifuge, fungicide, sudorific, vermifuge, and stimulant of leukocyte production [12,13,14]. Several ethnomedicinal surveys report its peculiar uses in different parts of Morocco. In the region of Rabat, it is utilized to treat metabolic diseases [15], in Agadir for diabetes [16], in Sidi-Boughaba as a stomachic, anthelmintic, antidiabetic, and anxiolytic medicine and as a treatment for nervous breakdowns and hepatic and gastric insufficiencies [13], and in Fez for digestive and neurological troubles [17].

**Figure 1:**
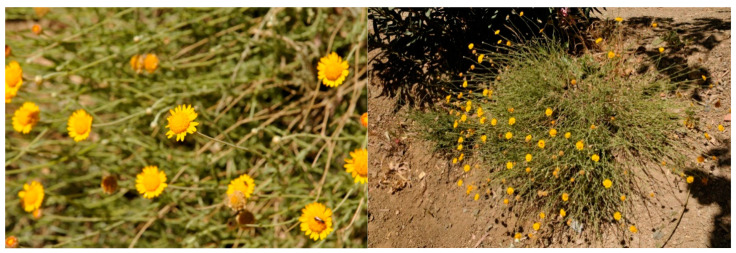
*Cladanthus scariosus* (Ball) Oberpr. & Vogt plants collected in Morocco.

*C. scariosus* (synonyms: *Chamaemelum scariosum* (Ball) Benedi; *Ormenis scariosa* (Ball) Litard & Maire, Basionym = *Santolina scariosa* Ball) is endemic to Morocco. It is a perennial plant with a strong aromatic odor, ranging from 30 to 60 cm in height; the stems are erect and ended by flower heads with orange-yellow ligules (Figure 1). This species is quite common in open areas on sandstone and is found in the Moroccan High Atlas [18].

*C. scariosus*, known in Morocco by several vernacular names (“*Irezghi*, *Irezgui*, *Itzghi*, *Ifskin’uarras*, *Gartûfa*”), is generally used to treat all disorders where spasms are important symptoms; it has tonic, stomachic, analgesic, and antispasmodic properties [18]. Furthermore, the tea of its leaves and inflorescences is used for gastrointestinal, gynecological, and pediatric troubles and for the treatment of diabetes [19,20].

Phytochemical investigation of the non-volatile constituents of *Cladanthus* species indicated the occurrence of flavonoids and phenolic acids in the extracts of *C. arabicus* [21] and *C. mixtus* [22,23], poliacetylenes in *C. arabicus* [24], coumarins in *C. mixtus* [25], and sesquiterpenes in *C. mixtus* [26] and *C. arabicus* [27].

Apart from *C. flahaultii*, which is totally devoid of any investigation, all the other four taxa have been studied for the composition of their essential oils (EOs) and for some biological activities as reported in Table 2.

These findings suggest that EOs derived from the *Cladanthus* species possess diverse biological properties, making them potentially valuable in various medical and therapeutic applications. For this reason, the aim of this work was to investigate the chemical composition and to evaluate the antiproliferative effect of the *C. scariosus* EO and its main compounds against the human breast adenocarcinoma cell line, human malignant melanoma cell line, and human colon adenocarcinoma cell line, three of the most common cancers today.

## 2. Results and Discussion

### 2.1. Chemical Composition of C. scariosus EO

Hydrodistilled *C. scariosus* EO, obtained from fully flowering aerial parts of the plant, had a dark blue color. Overall, forty-seven different compounds were identified and listed in Table 3.

The EO was found to be very rich in monoterpene hydrocarbons (43.0%) with *α*-pinene (11.9%), sabinene (10.2%), *p*-cymene (8.5%), and *α*-phellandrene (5.2%) as the most abundant compounds. However, the major constituent was germacrene D (13.2%), the principal constituent of sesquiterpene hydrocarbons (27.2%), whereas terpinen-4-ol (8.8%) was the principal component among the oxygenated monoterpenes (11.7%). Chamazulene, responsible for the blue color of the EO, was present at 4.0%. Fortunately, the response factor obtained from the appropriate calibration lines confirmed the percentage of *α*-pinene, *p*-cymene, germacrene D, and terpinene-4-ol compounds from the integrals of the areas of the GC-TOF/MS chromatogram (Table 2).

The data obtained in this work basically agreed with the compositions of the EOs from other accessions of *C. scariosus* previously published [18,48,49], although some differences should be noted. As can be seen from Table 2, the major compounds identified in *C. scariosus*, such as *α*-pinene, sabinene, chamazulene, and germacrene D (Table 3), are present in all the other accessions collected in different places in the Moroccan territory. They could be considered exactly as markers of the species in question.

The total composition detected also allows us to make comments about the chemical compositions of other *Cladanthus* ssp. EOs. Compounds such as sabinene, *α*-pinene, *p*-cymene, and terpinen-4-ol were similarly identified in *C. arabicus* EOs [28,29,30], but these did not present chamazulene nor germacrene D; differently, *C. eriolepis* EOs were rich in esters such as 2-methylbutyl angelate, 2-methylbutyl isobutyrate, and others [4,5], while the *C. mixtus* EOs present major compounds such as camphor, (*E*)-*β*-farnesene, and bornyl acetate [31,32,33,35].

### 2.2. Effects of C. scariosus EO on Tumor Cell Viability

**Figure 2:**
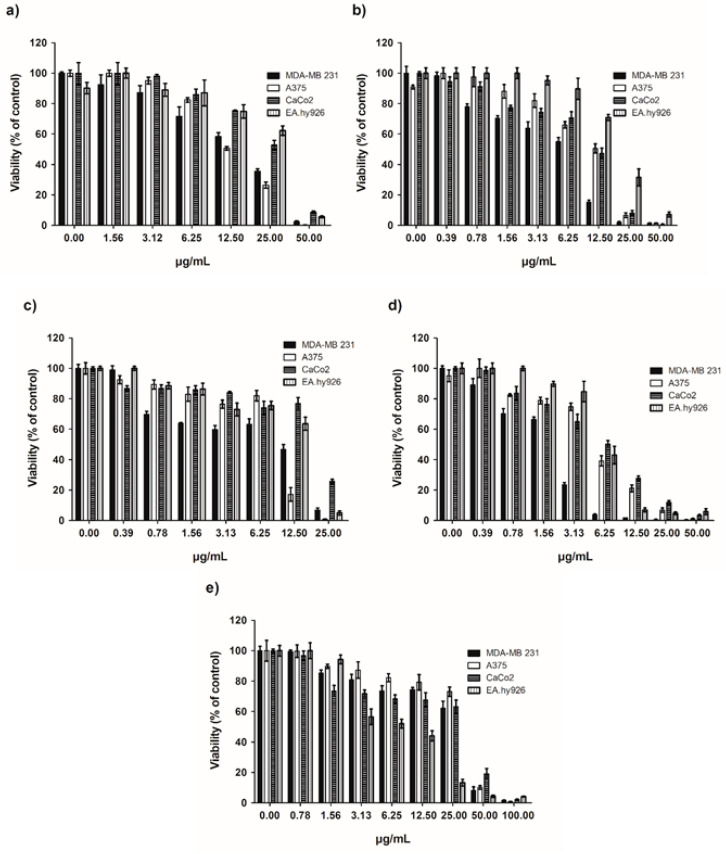
Cytotoxic effect of *C. scariosus* EO and its main compounds. Cell viability was determined in MDA-MB 231, A375, Caco2, and EA.hy926 cell lines by MTT. Cells were treated for 72 h with different concentrations of (**a**) *C. scariosus* EO, (**b**) germacrene D, (**c**) α-pinene, (**d**) terpinen-4-ol, and (**e**) *p*-cymene. Data shown are expressed as mean ± SE of three separate experiments.

In the literature, no data are available about the cytotoxic activity of *C. scariosus* EO. For the first time, we here report the effect of this EO against a panel of human cancer cells and a non-tumor cell line. Three human cancer cell lines, a human breast adenocarcinoma (MDA-MB 231), a human malignant melanoma (A375), and a human colon adenocarcinoma (CaCo2), and one human endothelial cell line (EA.hy926) were treated with different concentrations of EO and its main compounds such as germacrene D, α-pinene, terpinen-4-ol, and *p*-cymene for 72 h. An MTT assay was used to evaluate the cytotoxic activity of EO, and the results obtained are shown in Table 4 and Figure 2.

*C. scarious* EO showed antiproliferative activity against all three cell lines with IC_50_ values of 13.69, 13.21, and 22.71 µg/mL for MDA-MB 231, A375, and CaCo2, respectively. Based on the criteria of the American National Cancer Institute for considering a crude extract promising for further purification, the IC_50_ value being lower than 30 μg/mL [50] suggests that the EO shows excellent activity against tumor cells. Such a cytotoxic activity of EO could also be attributed to its main components. Indeed, in our experiments, germacrene D and terpinen-4-ol were the most active on all three cell lines, with IC_50_ values ranging from 3.21 to 5.23 µg/mL for terpinen-4-ol and from 4.41 to 8.57 µg/mL for germacrene D on MDA-MB 231 and CaCo2, respectively.

Noteworthy, the cytotoxic activity of terpinen-4-ol is comparable with that of cisplatin, showing IC_50_ values of 2.54 and 3.07 µg/mL on MDA-MB 231 and CaCo2 cell lines, respectively. Germacrene D showed IC_50_ values slightly higher than IC_50_ values for terpen-4-ol, and α-pinene showed remarkable activity on MDA-MD 231 (IC_50_ value, 12.40 µg/mL; 91 µM) and on A375 (IC_50_ value 17.04, 125 µM). *p*-Cymene exerted lower cytotoxic activity. The data are in good agreement with the reported antiproliferative activity of terpinen-4-ol against the human acute promyelotic leukemia cell line HL60 [51], human non-small-cell lung cancer cell line HNCLC [52], and human leukemic MOLT-4 cell line [53], where terpinen-4-ol induced apoptotic and macrophagic cell death. Terpinen-4-ol was active on melanoma, as reported in the literature [54]; it potently induces cell cycle arrest, apoptosis, and necrotic cell death [55]. Germacrene D was reported to be active on human breast adenocarcinoma (MDA-MB 231 and MCF-7), human ductal carcinoma (Hs578T), and human hepatocellular carcinoma (HepG2) [56]. α-Pinene has also been reported to exhibit apoptotic and antimetastatic activity on melanoma cells [57]. In the literature, sabinene also exerts cytotoxic activity against liver (HepG2), colon (HCT116), and breast (MCF7) cancer cell lines [58]. From the data reported above on the antiproliferative activities of the main compounds of the EO, it could be hypothesized that the EO activity may involve the activation of apoptotic processes, a study project that could be explored further later.

Although the presence of germacrene D (13.2%), α-pinene (11.9%), sabinene (10.2%), terpinen-4-ol (8.8%), and *p*-cymene (8.5%) may explain the cytotoxic activity of *C. scariosus* EO, it cannot be excluded that even the presence of lower-concentration components might contribute to the final cytotoxic activity observed.

Although the cytotoxic activity of the essential oil is significantly relevant to tumor cell lines, its specificity is not very high. In fact, its cytotoxic activity on the normal vascular endothelial line EA.hy926 is only slightly lower than that of MDA-MB 231 and A375. Perhaps a better comparison can be made with a normal cell line of ectodermal derivation given that the three tumor lines tested are all of epithelial origin.

## 3. Materials and Methods

### 3.1. Plant Material

Aerial parts from several fresh individuals of *C. scariosus*, at the full flowering stage, were collected near Tizi n’Ticha, Morocco, at about 2170 m a.s.l., 31°17′18″ longitude N and 7°22′55″ latitude W, in May 2023. One of the samples, identified by Prof. Vincenzo Ilardi, has been stored in the University of Palermo Herbarium (No. PAL 109762).

### 3.2. Isolation of EO

**Figure 3:**
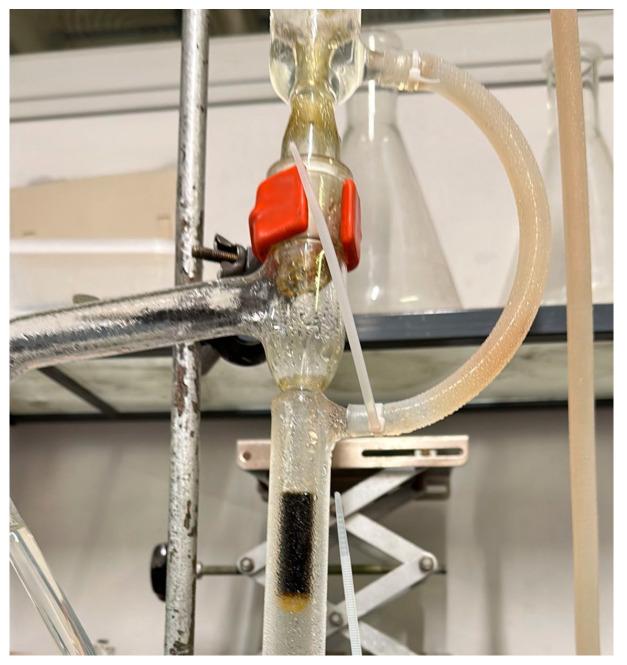
Close-up photo of *C. scariosus* EO isolated by the Clevenger apparatus.

Fresh aerial parts (660 g) were subjected to hydrodistillation for 3 h, according to the standard procedure described in European Pharmacopoeia [59]. The EO was dried over anhydrous Na_2_SO_4_ and preserved at 4 °C prior to further analysis (up to one month). Samples yielded 0.15% of EO. Hydrodistilled *C. scariosus* EO, obtained from fully flowering aerial parts of the plant, had a dark blue color (Figure 3).

### 3.3. GC-MS Analysis and Quantitative Determination

Analysis of EOs was carried out according to the procedure reported by Lauricella et al. [60]. The composition of the EO was determined by GC–MS analyses. They were achieved on an Agilent Technologies 7890 GC equipped with FID and mass spectrometer detectors using a DB-5MS (5% phenylmethylpolysiloxane) capillary column (30.00 m × 0.25 mm, 0.25 μm film thicknesses; J & W Scientific, Folsom, CA, USA). The carrier gas was helium at a flow rate of 0.8 mL/min. The initial column temperature was 60 °C and programmed to increase up to 280 °C at a rate of 4 °C/min. The split ratio was 40:1. The injector temperature was set at 300 °C. The acquisition range was 50–550 *m*/*z* in electron-impact (EI) mode using an ionization voltage of 70 eV. The EO was diluted 1:100 in *n*-hexane, and then 0.1 μL was injected into the GC system.

Six calibration solutions containing α-pinene, terpinen-4-ol, germacrene D, and *p*-cymene were prepared in hexane at a concentration (*w/w* %) of 0.5, 1.0, 2.0, 5.0, 10.0, and 20.0%. These calibration solutions contained the compounds, except for the unavailable sabinene constituent, present in greater abundance in the EO sample. Linear retention indices (LRIs) were calculated using a mixture of pure *n*-alkanes (C_8_–C_40_), and all the peaks’ compounds were identified by comparison with MS and by comparison of their relative retention indices with WILEY275, NIST 17, ADAMS, and FFNSC2 libraries.

### 3.4. Pure Compounds

Pure compounds, such as α-pinene, terpinen-4-ol, and *p*-cymene, were purchased from Sigma-Aldrich (Sigma-Aldrich Chemie GmbH, Eschenstr. 5, 82,024 Taufkirchen, Germany), whereas germacrene D was isolated from the essential oil of *Kundmannia sicula* (L.) DC. [61].

### 3.5. Cell Culture

Cytotoxic studies were determined in A375 (human malignant melanoma cell line), MDA-MB 231 (human breast adenocarcinoma cell line), CaCo2 (human colon adenocarcinoma cell line), and EA.hy926 (human vascular endothelial cells). A375, MDA-MB 231, and EA.hy926 were grown in Dulbecco’s Modified Eagle’s Medium (DMEM) with 2 mM L-glutamine, 100 IU/mL penicillin, and 100 µg/mL streptomycin and was supplemented with 10% heat-inactivated fetal bovine serum (HI-FBS). CaCo2 was cultured in DMEM medium with 2 mM L-glutamine, 100 IU/mL penicillin, 100 μg/mL streptomycin, and 1% Non-Essential Amino Acid (NEAA) and was supplemented with 10% HI-FBS. Cells were maintained in a humidified atmosphere with 5% CO_2_ at 37 °C.

### 3.6. MTT Assay

Cell viability was examined by the ability of the cells to cleave the tetrazolium salt MTT [3-(4,5-dimethyl-2-thiazolyl)-2,5-diphenyl-2H-tetrazoliumbromide] using the mitochondrial enzyme succinate dehydrogenase following the procedure described earlier [62]. Briefly, cells were seeded at a density of 2 × 10^4^ cells/mL. After 24 h, samples were exposed to different concentrations of essential oil and standards (0.78–200 µg/mL) and incubated for 72 h in a humidified atmosphere of 5% CO_2_ at 37 °C. The anticancer drug cisplatin (0.01–50 μg/mL) was used as the positive control. At the end of incubation, each well received 10 µL of MTT (5 mg/mL in phosphate-buffered saline, PBS) and the plates were incubated for 4 h at 37 °C. Then, the supernatant was removed, and DMSO (dimethyl sulfoxide) was added to dissolve the formazan crystals. The plates were placed on a shaker for 15 min and the optical density was determined at 540 nm using a microplate spectrophotometer FLUOstar Omega (BMG Labtech, Milan, Italy). Experiments were conducted in triplicate. The cell survival curves were calculated after comparing with the vehicle (Et-OH). The 50% cytotoxic concentration (IC_50_) was defined as the compound concentration required to reduce the cell viability by half. The IC_50_ values were determined with the GraphPad Prism 5 computer program (GraphPad Software, San Diego, CA, USA).

## 4. Conclusions

In this study, the chemical profile of *C. scariosus* EO’s aerial parts was examined. The most abundant class was monoterpene hydrocarbons (43.0%), in which the main compounds were *α*-pinene (11.9%), sabinene (10.2%), *p*-cymene (8.5%), and *α*-phellandrene (5.2%). This composition was found to be very similar to the other five Moroccan *C. scariosus* accessions, not only in terms of the main components but also the different sub-divisions of metabolite classes. To explore the anticancer potential of the EO and its main components, their effects on several cancer cell lines were tested. The remarkable cytotoxic activity is promising for use in cancer prevention and treatment or in combination with conventional chemotherapy drugs to reduce the toxicity of the latter. Further in vitro and in vivo studies on the anticancer mechanisms exerted by EO and the components are required to validate these preliminary data. In addition, studies are necessary to demonstrate the non-toxicity of EO on non-tumor cells as well as the elucidation of the molecular mechanisms governing the anticancer properties of this EO and their major constituents.

## Figures and Tables

**Table 1:** Distribution and synonymous of all the taxa of genus *Cladanthus.*

Taxa	Synonymous	Distribution
*Cladanthus arabicus* (L.) Cass.	*Anthemis arabica* L. *Chamaemelum cladanthus* E.H.L.Krause	Morocco, Algeria, Tunisia, Lybia, Malta, Spain
*Cladanthus eriolepis* (Maire) Oberpr. & Vogt	*Chamaemelum eriolepis* (Maire) Benedì *Ormenis eriolepis* Maire	Morocco
*Cladanthus flahaultii* (Emb.) Oberpr. & Vogt	*Chamaemelum flahaultii* (Emb.) Benedí *Ormenis flahaultii* Emb.	Morocco
*Cladanthus mixtus* (L.) Chevall.	*Anthemis mixta* L. *Chamaemelum mixtum* (L.) All. *Ormenis mixta* (L.) Dumort. *Ormenis aurea* Durieu *Ormenis multicaulis* Braun-Blanq. & Maire	All Mediterranean countries
*Cladanthus scariosus* (Ball) Oberpr. & Vogt	*Chamaemelum scariosum* (Ball) Benedí *Ormenis scariosa* (Ball) Litard. & Maire *Santolina scariosa* Ball	Morocco

**Table 2:** Main constituents (>3%) and several biological properties of the EOs of all the taxa of *Cladanthus* studied so far.

Taxa	Origin	P.	Compounds (%)	Biological Properties Investigated	Ref.
*C. arabicus*	Ourika, Morocco	ap	sabinene (31.1), *β*-pinene (16.7), *β*-myrcene (12.3), α-pinene (5.3), *cis*-chrysanthenyl acetate (3.4)	antioxidant, antimicrobial, insecticidal	[28]
	Ouarzazate, Morocco	ap	sabinene (13.3), *β*-pinene (12.8), *α*-phellandrene (8.7), *β*-myrcene (6.9), α-pinene (8.3), *p*-cymene (4.4)	antibacterial	[29]
	Ouarzazate, Morocco	fl	*β*-pinene (31.9), sabinene (22.9), *p*-cymene (8.3), α-pinene (5.0), *epi*-*α*-cadinol (5.0), terpinen-4-ol (3.6)		
	Agadir, Morocco	ap	*β*-pinene (23.6), t-cadinol (9.5), diethyl phthalate (7.9), α-pinene (5.7)		[30]
*C. eriolepis*	Errachidia, Morocco	ap	camphor (37.0), sabinene (10.3), *α*-pinene (6.3), *p*-cymene (6.1), *α*-cadinol (5.6), tricyclene (3.8), artemisia ketone (3.4),	antibacterial	[6]
	Errachidia, Morocco	ap	*α*-pinene (13.0), isobutyl angelate (10.7), 2-methylbutyl angelate (9.5), germacrene D (7.1), sabinene (4.4)	antioxidant, antifungal	[4]
	Errachidia, Morocco	l	isobutyl angelate (23.9), 2-methylbutyl angelate (20.0), isobutyl isobutyrate (8.7), 2-methylbutyl isobutyrate (6.5), *β*-bisabolene (5.2), 2-methylbut-2-en-1-yl acetate (4.3), methyl allyl angelate (3.3)		
	Errachidia, Morocco	fl	*β*-bisabolene (36.0), isobutyl angelate (12.2), isobutyl isobutyrate (7.6), 2-methylbutyl angelate (5.9), *γ*-curcumene (4.2), 2-methylbut-2-en-1-yl acetate (3.3), 2-methylbutyl isobutyrate (3.0)		
	Jbal Zagora, Morocco	ap	isobutyl angelate (22.0), isobutyl isobutyrate (21.2), *α*-pinene (9.2), 2-methylbutyl angelate (7.7), 2-methylbutyl isobutyrate (5.7), 2-methylallyl isobutyrate (5.3), 2-methylallyl angelate (4.6)		[5]
	Ouled Ouchah, Morocco	ap	isobutyl angelate (22.4), isobutyl isobutyrate (20.8), *α*-2-methylbutyl angelate (7.2), pinene (5.8), 2-methylbutyl isobutyrate (5.8), 2-methylallyl isobutyrate (5.5), 2-methylallyl angelate (4.9), *β*-bisabolene (4.0), (*E*)-2-methylbutenyl propionate (3.0)		[5]
*C. mixtus*	Algeria	ap	*α*-thujone (51.8), *β*-thujone (14.6), borneol (7.3), 3-hexenol (4.9)	antibacterial	[31]
	Temara, Morocco	ap	camphor (21.4), *β*-myrcene (13.8), santolinatriene (10.1), santolina alcohol (5.4), *α*-pinene (4.4), yomogi alcohol (4.1), camphinelone (3.8), 1,8-cineol (3.1), *cis*-*α*-bisabolene (3.0), (*E*)-*β*-farnesene (3.0)		[32]
	Tamensa, Morocco	fl	camphor (17.5), (*E*)-*β*-farnesene (13.8), (*E*,*E*)-*α*-farnesene (5.6), germacrene D (5.0), *cis-β*-ocimene (4.9), bornyl acetate (4.6), *β*-myrcene (4.4), santolinatriene (3.2)		[33]
	Tamensa, Morocco	l	camphor (19.9), bornyl acetate (5.5), (*E*)-*β*-farnesene (4.5), lavandulyl acetate (4.2), borneol (3.3), germacrene D (3.2)		[33]
	Sidi Allai Lbahraoui, Morocco	fl	(*E*)-*β*-farnesene (8.3), ledol (5.5), *α*-bisabolol (3.2)		[33]
	Sidi Allai Lbahraoui, Morocco	l	(*E*)-nerolidol (44.1), ledol (6.9), (*E*)-*β*-farnesene (5.1)		[33]
	Settat, Morocco	fl	camphor (29.0), *β*-myrcene (11.6), *cis*-chrysanthenyl acetate (4.6), camphene (4.1), (*E*)-*β*-farnesene (3.3), bicyclogermacrene (3.0)		[33]
	Settat, Morocco	l	*β*-myrcene (12.4), camphor (9.4), bicyclogermacrene (6.1), (*E*)-*β*-farnesene (3.6), bornyl acetate (3.0)		[33]
	Kenitra, Morocco	fl	camphor (14.4), neryl acetate (8.1), bornyl acetate (4.7), (*E*)-*β*-farnesene (4.5), *trans*-*α*-nerodyl acetate (3.1)		[33]
	Kenitra, Morocco	l	camphor (11.0), hexadecanoic acid (8.6), bornyl acetate (7.6), neryl acetate (6.0), spathulenol (3.6), (*E*)-nerolidol (3.2)		[33]
	Benguerir, Morocco	fl	2-tridecanone (21.5), camphor (17.8), *n*-pentacosane (9.5), (*E*)-*α*-bisabolene (6.7), bornyl acetate (3.4)		[33]
	Benguerir, Morocco	l	camphor (33.0), 2-tridecanone (7.8), 2-pentyl-1furan (4.4)		[33]
	Mamora, Morocco	ap	santolina alcohol (37.7), *α*-pinene (4.8), camphenilone (4.8), yomogi alcohol (4.5), germacrene (3.3), 1,8-cineole (3.2), cubenol (3.2)	antibacterial	[34]
	Morocco	ap	bornyl acetate (45.0), limonene (10.0), *α*-pinene (8.0)		[35]
	Morocco		santolina alcohol (32.0), *α*-pinene (15.0), limonene (8.0), germacrene (5.0)		[36]
	Morocco		santolina alcohol (37.0), *α*-pinene (16.0), limonene (6.0)		[37]
	Benguir, Morocco	ap	camphor (26.8), santolina triene (9.1), camphene (8.1), 2-tridecanone (6.3), *β*-myrcene (5.1), *α*-thujene (5.1), *β*-pinene (4.3), palmitic acid (3.3)		[38]
	Kenitra, Morocco	ap	camphor (25.8), *β*-myrcene (17.4), camphene (7.4), santolina triene (7.2), *α*-pinene (3.7), *trans*-*β*-farnesene (3.3), sabinene (3.0)		[38]
	Settat, Morocco	ap	camphor (18.8), *β*-myrcene (10.2), camphene (6.0), germacrene D (5.3), *β*-pinene (4.7), *trans*-*β*-farnesene (3.8)		[38]
	Meknes, Morocco	ap	camphor (20.9), santolina triene (10.3), *β*-myrcene (9.1), *trans*-*β*-farnesene (7.9), camphene (5.0), *trans*-*α*-nerodyl acetate (4.1), *α*-pinene (3.3)		[38]
	Tamesna, Morocco	ap	santolina triene (15.3), camphor (13.6), *trans*-*β*-farnesene (8.5), *trans*-*α*-nerodyl acetate (5.6), camphene (4.5), bornyl acetate (3.9)		[38]
	Chefchaouane, Morocco	ap	*β*-myrcene (26.5), *trans*-*β*-farnesene (17.9), 2-tridecanone (15.5), camphor (10.0), germacrene D (8.6)		[38,39]
	Oujda, Morocco	ap	*trans*-*β*-farnesene (42.9), dendrolasin (3.0)		[38,39]
	Bouznika, Morocco	ap	2-methyl-2-*trans*-butenyl methacrylate (34.0), *ar*-curcumene (13.6), pinocarvone (5.6), *β*-myrcene (3.0)		[38]
	Sidi Alal Ibahraoui, Morocco	ap	santolina alcohol (17.4), 1,8-cineole (11.6), *β*-elemene (5.0), sabinene (3.9)		[38]
	Sicily, Italy	ap	hexadecanoic acid (15.2), santolina alcohol (11.7), *τ*-cadinol (6.7), spathulenol (6.5), caryophyllene oxide (3.8), artemisia alcohol (3.8), *τ*-muurolol (3.0)	antibacterial	[40]
	Sicily, Italy	r	hexadecanoic acid (52.0), 1,2-benzenedicarboxylic acid bis(2-methylpropyl) ester (12.3), tetradecanoic acid (4.8), dodecanoic acid (4.2), hexahydrofarnesylacetone (3.0)		
	Commercial, Morocco		*α*-pinene (11.5), santolina alcohol (10.2), *trans*-*β*-farnesene (8.6), 1,8-cineole (7.4), germacrene D (6.3), *δ*-elemene (3.6)		[41]
	Kenitra, Morocco	ap	santolina alcohol (27.3), germacrene D (10.2), (*E*)-*β*-farnesene (4.5), *α*-pinene (4.5), *epi*-*α*-cadinol (4.4), *α*-cadinol (3.8), artemesia alcohol (3.6), caryophylladienol (3.1)		[9]
	Mezraoua, Morocco MWE	ap	santolina alcohol (40.7), germacrene D (8.9), α-pinene (5.7), artemesia alcohol (4.3), (*E*)-*β*-farnesene (4.0), limonene (3.1), yomogi alcohol (3.0)	antioxidant, antimicrobial	[42]
	Sardinia, Italy	ap	santolina alcohol (46.2–39.8), (*Z*)-heptadeca-9,16-dien-7-one (12.7–12.3), (*E*)-*β*-farnesene (5.6–3.0), artemisia alcohol (4.7–3.4), α-pinene (3.5–0.6)	antimicrobial	[43]
	Corsica, France Claster 2	ap	yomogi alcohol (16.2–14.3), santolina alcohol (15.6–12.5), artemisia alcohol (13.2–12.0), (*Z*)-heptadeca-9,16-dien-7-one (10.7–9.9), germacrene D (9.0–7.0), (*E*)-*β*-farnesene (6.8–5.2), (*E*,*E*)-α-farnesene (6.6–6.5)	antimicrobial	[43]
	Corsica, France Claster 3	ap	germacrene D (28.6–13.3), santolina alcohol (26.2–15.4), (*E*,*E*)-α-farnesene (15.6–3.4), (*Z*)-heptadeca-9,16-dien-7-one (11.5–4.4), (*E*)-*β*-farnesene (11.3–1.9), yomogi alcohol (6.4–07), α-pinene (3.9–1.0)	antimicrobial	[43]
	Taounate, Marocco	ap	santolina alcohol (37.5), (*E*)-*β*-farnesene (6.2), *epi*-α-muurulol (5.0), α-cadinol (4.9), α-pinene (4.3), artemisia alchol (4.1), germacrene D (3.8), 1,8-cineole (3.2), campor (3.0)	antimicrobial	[44]
	Taounate, Morocco	ap	p-menthane-1,8-diol (18.2), α-pinene (10.8), germacrene D (9.2), (*Z*)-*β*-farnesene (7.6), *δ*-elemene (7.2), caryophillene (3.6), *β*-elemene (3.3), *β*-pinene (3.2), α-muurolene (3.0)	antibacterial	[45]
	Taounate, Morocco	ap	germacrene D (11.5), 1,8-cineol (10.3), *cis*-methyl-eugenol (9.0), butyric acid (8.5), *δ*-elemene (5.5), *cis*-cadina-1(6),4-diene (3.2)	antioxidant, antibacterial	[46]
	Morocco	fl	α-pinene (24.9), santolina alcohol (15.2), 1,8-cineole (7.1), elemene (6.7), limonene (6.0), *α*-humulene (4.1), myrcene (3.7), sabinene (3.4)	insecticidal	[47]
*C. scariosus*	Clade 1 Morocco	ap	sabinene (17.8), *α*-pinene (14.3), germacrene D (9.5), dihydrochamazulene (7.5), chamazulene (4.4), *α*-phellandrene (4.2), terpinen-4-ol (3.9)		[18]
	Clade 2 Morocco	ap	sabinene (23.7), *α*-pinene (16.0), terpinen-4-ol (8.2), dihydrochamazulene (6.4), germacrene D (4.9), *γ*-terpinene (4.7), chamazulene (3.0)		[18]
	Clade 3 Morocco	ap	*α*-pinene (13.7), sabinene (11.0), terpinen-4-ol (7.5), germacrene D (5.6), camphor (4.8), *α*-phellandrene (3.5), chamazulene (3.2)		[18]
	Oukeimeden Morocco	ap	germacrene D (20.7), (*E*)-chrysanthenyl acetate (8.3), chamazulene (7.1), sabinene (6.9), *α*-pinene (4.8), *t*-muurolol (4.2), (*E*,*E*)-farnesyl acetate (3.9)		[48]
	Ait M’hamed Morocco	ap	*p*-cymene (11.3), caryophyllene oxide (9.9), *γ*-terpinene (8.3), sabinene (7.1), elemol (6.5), *α*-terpinolene (5.6), spathulenol (5.0), germacrene D (4.9), *α*-pinene (3.5), camphor (3.0), chamazulene (1.6)	antioxidant, antimicrobial	[49]

P. = parts; ap = aerial parts; fl = flowers; l = leaves; r = roots.

**Table 3:** Chemical composition (%) of *Cladanthus scariosus* essential oil (EO) collected in Morocco.

No.	Compound ^a^	LRI ^b^	LRI ^c^	A (%) ^d^	Content ^e^ (*w*/*w*%)
1	*α*-Thujene	933	927	1.4	
2	*α*-Pinene	942	933	11.9	11.42
3	Camphene	958	953	0.1	
4	Thuja-2,4(10)-diene	961	953	0.1	
5	Benzaldehyde	970	960	0.1	
6	2-Methylbutyl-propanoate	973	968	0.2	
7	Sabinene	980	972	10.2	
8	*β*-Pinene	986	978	0.6	
9	Myrcene	992	991	0.2	
10	*α*-Phellandrene	1012	1007	5.2	
11	*α*-Terpinene	1022	1018	1.1	
12	*p*-Cymene	1030	1025	8.5	8.46
13	Limonene	1034	1030	0.5	
14	*β*-Phellandrene	1036	1031	0.3	
15	1,8-Cineole	1038	1032	0.4	
16	Isopentyl-butyrate	1056	1054	0.1	
17	*γ*-Terpinene	1062	1058	2.3	
18	*n*-Octanol	1071	1076	0.2	
19	Terpinolene	1090	1086	0.6	
20	Linalool	1101	1101	1.5	
21	Limona ketone	1135	1131	0.2	
22	Borneol	1178	1173	0.3	
23	Terpinen-4-ol	1187	1184	8.8	8.91
24	*α*-Terpineol	1199	1195	0.5	
25	*δ*-Elemene	1337	1335	1.0	
26	*α*-Cubebene	1352	1347	0.1	
27	*α*-Ylangene	1376	1371	tr	
28	*α*-Copaene	1383	1375	0.9	
29	*β*-Bourbonene	1391	1382	0.3	
30	*β*-Elemene	1394	1390	0.6	
31	Methyl eugenol	1401	1403	0.4	
32	*β*-Ylangene	1427	1422	2.2	
33	(*E*)-Caryophyllene	1429	1424	2.1	
34	*β*-Copaene	1438	1433	1.5	
35	Isogermacrene D	1453	1447	0.6	
36	*α*-Humulene	1464	1454	0.4	
37	Germacrene D	1490	1480	13.2	12.98
38	Viridiflorene	1500	1491	0.2	
39	*γ*-Amorphene	1500	1490	0.2	
40	Bicyclogermacrene	1505	1497	1.1	
41	*ε*-Amorphene	1509	1502	0.7	
42	*γ*-Cadinene	1521	1512	0.7	
43	*δ*-Cadinene	1525	1518	1.4	
44	(*Z*)-Cadin-4en-7-ol	1647	1638	3.2	
45	*δ*-Cadinol	1651	1641	1.2	
46	Cadin-4-en-10-ol	1666	1659	1.5	
47	Chamazulene	1742	1732	4.0	
	**Monoterpene Hydrocarbons**			**43.0**	
	**Oxygenated Monoterpenes**			**11.7**	
	**Sesquiterpene Hydrocarbons**			**27.2**	
	**Oxygenated Sesquiterpenes**			**5.9**	
	**Hydrocarbons**			**4.0**	
	**Phenylpropanoids**			**0.4**	
	**Other Compounds**			**0.6**	
	**Total Composition**			**92.8**	

^a^ Components listed in order of elution on a DB-5 MS column; ^b^ linear retention indices on a DB-5 MS non-polar column; ^c^ linear retention indices based on the literature (https://webbook.nist.gov/; accessed on 15 March 2024); ^d^ percentage amounts of the separated compounds calculated from integration of the peaks; ^e^ the content is the gravimetric percentage of α-pinene, terpinen-4-ol, germacrene D, and *p*-cymene determined using appropriate calibration lines.

**Table 4:** In vitro cytotoxic activity of *C. scariosus* EO and its main constituents.

	Cell Line (IC_50_ µg/mL) ^a^			
	MDA-MB 231 ^b^	A375 ^c^	CaCo2 ^d^	EA.hy926 ^e^
Essential oil	13.69	13.21	22.71	24.51
95% C.I. ^f^	12.48–13.99	11.98–15.65	20.06–25.72	20.92–28.70
Germacrene D	4.41	9.53	8.57	17.85
95% C.I.	3.75–5.18	8.32–10.92	7.25–10.12	16.28–19.57
α-Pinene	12.40	17.04	30.94	22.14
95% C.I.	8.79–17.49	14.88–19.52	25.68–37.28	18.07–27.13
Terpinen-4-ol	3.21	4.91	5.23	5.57
95% C.I.	2.79–3.70	4.34–5.54	4.66–5.89	5.13–6.07
*p*-Cymene	29.57	27.48	27.90	6.63
95% C.I.	26.91–32.49	23.53–32.09	26.64–29.21	5.53–7.98
**Positive control**				
Cisplatin	2.54	0.62	3.07	ND
95% C.I.	1.82–2.74	0.55–0.70	2.15–3.92	

^a^ IC_50_ = The concentration of compound that affords a 50% reduction in cell growth (after 72 h of incubation). ^b^ Human breast adenocarcinoma cell line. ^c^ Human malignant melanoma cell line. ^d^ Human colon adenocarcinoma cell line. ^e^ Human vascular endothelial cells. ^f^ Confidence interval. ND: not determined.

## Data Availability

All data and materials are available upon request from the corresponding author.
